# 

**Published:** 1902-03-08

**Authors:** 


NOTICE TO CORRESPONDENTS.

All MSS., Letters, Books for Review, and other matters intended for the Editor should be addressed The Editor, "The Hospital," The Hospital Building, 28 & 29 Southampton Street, Strand, W.C.
The Editor cannot undertake to return rejected MS., even when accompanied by stamped directed envelope.

**Notice to Advertisers and Subscribers.**

All Advertisements, Orders for Copies of The Hospital, and Business Communications should be addressed to **THE MANAGER (Not to the Editor),** The Hospital, 28 & 29 Southampton Street, Strand, London, W.C.

The Cost of Subscription, post free, is as follows:—Per week, 2½d.; per quarter, 2s. 8d.; per half-year, 5s. 3d.; per annum, 10s. 6d. Foreign:—Per annum, 15s. 2d., payable, in all cases, in advance.

**NOTICE.—Vol. XXX. of "The Hospital" (April 1901, to September 1901), in handsome cloth binding can now be obtained at the office of this Journal, price, 7s. 6d. Cases for binding the Numbers contained in this Volume 1s. 6d. Index to Vol. XXX., 2½d., post free.**

**The Monthly Part for March is now ready. Price 6d., by post 8d.**

*NOW READY.*

**BURDETT'S HOSPITALS & CHARITIES, 1902.**

**The Year-book of Philanthropy and Hospital Annual.**

**By Sir HENRY BURDETT.**

Thirteenth Year. Crown 8vo., over 1,000 pp.
Cloth, **5/-** post free.

THE SCIENTIFIC PRESS, LIM., 28 & 29 Southampton Street, Strand, London, W.C.

